# Pulmonary Vein Stenosis: Incremental Knowledge Gains to Improve Outcomes

**DOI:** 10.3390/children8060481

**Published:** 2021-06-07

**Authors:** Rachel D. Vanderlaan, Christopher A. Caldarone

**Affiliations:** 1 Division of Cardiovascular Surgery, Hospital for Sick Children, Toronto, ON M5G 1X8, Canada; 2 Division of Cardiovascular Surgery, Texas Children’s Hospital, Houston, TX 77030, USA; Cacaldar@texaschildrens.org

**Keywords:** pulmonary vein stenosis, pediatric, pathophysiology

## Abstract

Pulmonary vein stenosis remains a considerable clinical challenge, with high mortality still present in children with progressive disease. In this review, we discuss the clinical spectrum of pulmonary vein stenosis and what is known about the etiology and potential modifying and contributing factors in progressive pulmonary vein stenosis.

## 1. Introduction

Pulmonary vein stenosis (PVS) is a rare pediatric vascular disease characterized by progressive neointimal lesion and associated with high mortality. The inherent clinical heterogeneity of patients, unclear etiology and progressive pathophysiology impact on our ability to make substantial progress in improving outcomes for children with PVS. In this review, we discuss barriers to clinical research for children with PVS, our current state of knowledge regarding mechanisms contributing to the development of PVS and research priorities moving forward.

## 2. Clinical Research: Not All Pulmonary Vein Stenosis Is Created Equal

Rare pediatric diseases are inherently difficult to both treat and study due to small patient numbers, dispersed expertise and limited funding opportunities. Pulmonary vein stenosis is no exception, and the variability in clinical spectrum of PVS further compounds our ability to advance clinical care and research. Clinical variability exists in the classification or type of PVS, pulmonary vein involvement, and comorbidities of patients and within the research realm, this can translate into ill-defined inclusion criteria and outcome measures. 

PVS can be classified as ‘primary PVS’ (or congenital), whereby the veins are normally connected to the left atrium, or ‘post repair PVS’, where the pulmonary veins drained anomalously, and PVS develops after veins have been surgically connected to the left atrium [1]. An important special population are children who are ex-premature and develop PVS, as their disease trajectory and comorbidities may be unique [2]. One could also add in-stent stenosis as another potential subcategory, where the etiological mechanism of stenosis (mitogenic stimulus due to radial forces) may be different from the original PVS that was treated by the stent. ‘Acquired’ PVS describes PVS caused by other factors such as radiofrequency ablation and fibrosing mediastinitis. 

The absence of definitive diagnostic criteria for PVS and thresholds for interventions can leave clinicians feeling uncertain around treatment plans. Generally, echocardiographic data demonstrating mean pressure gradients ≥4 mmHg, in the absence of flow redistribution, and with supporting anatomical evidence of stenosis by other imaging modalities, are typically considered significant PVS [3,4]. Supporting evidence of right ventricular pressure overload confirms hemodynamically significant pulmonary vein stenosis. Typically, both physiological evidence by echo and anatomical data through axial imaging or diagnostic angiogram confirm a diagnosis and allow for individualization of treatment plans.

**Figure 1:**
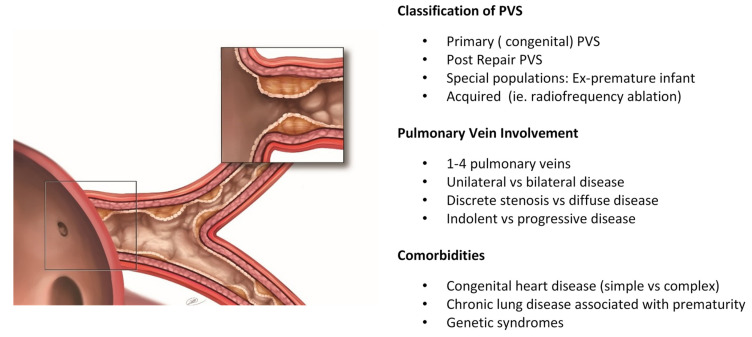
Variability in the clinical spectrum of pulmonary vein stenosis. PVS, pulmonary vein stenosis (Reprint permission, RD Vanderlaan, PVS Network, www.PVSNetwork.org, accessed on 1 May 2021).

PVS can also present with considerable variability in patients as it can occur in one or more of the pulmonary veins and can be bilateral or limited to unilateral disease. Lesions in the pulmonary veins can be discrete at the venoatrial junction or can be diffuse, extending along the length of the intraparenchymal vein (Figure 1), and the velocity at which the disease progresses can range from indolent to aggressive and relentless. From small cohort studies, markers of advanced disease such as multivessel involvement, bilateral disease and high preoperative right ventricular systolic pressure are associated with increased mortality [5,6,7], while imaging studies investigating characteristics of upstream pulmonary veins, as a marker of advanced disease, suggest that a smaller cross-sectional area in upstream veins was associated with increased risk of death [8]. 

Another layer of complexity is the important comorbidities that children may have in addition to PVS. Children with PVS may also have concomitant congenital heart disease (simple or complex) or chronic lung disease due to prematurity, all of which will affect clinical decision making. In addition, as many as 30% have some identifiable genetic syndrome (trisomy 21, CHARGE, Smith-Lemli-Opitz), which may have additional cardiac and extracardiac abnormalities [6,9,10]. Treatment of PVS must incorporate patient specific comorbidities to help improve outcomes. 

### Clinical Priorities

At the clinical level, creation of an institutional multidisciplinary PVS team is critical to overcoming the challenges with diagnostic and intervention thresholds and allows consensus for disease surveillance [1,11]. At a multi-institutional level, the PVS Network was formed in 2015 and is a collaborative community that seeks to improve clinical care and research for children with PVS (www.PVSNetwork.org, accessed on 1 May 2021). The PVS Network Registry will help to overcome sample size issues as it collects data across 19 institutions to provide insights into clinical practice and disease surveillance. Multidisciplinary collaboration will help to provide consensus for diagnosis criteria, descriptive anatomical nomenclature and identifying relevant outcome measures that will inform research studies. With over 600 patients currently enrolled, research targeting special subpopulations will provide insights into etiology, risk factors and outcomes. The PVS Network Registry studies will provide an exciting opportunity to address high priority knowledge gaps and provide data to inform consensus practice guidelines. 

## 3. Translating Molecular Understanding into Clinical Action 

The clinical spectrum of PVS will benefit from reframing as our understanding grows from emerging molecular mechanisms of PVS and potential contributing factors. Further translational and basic science research is needed to provide insights into the etiology and potential therapeutic approaches to PVS. 

### 3.1. Neointimal Lesions in PVS 

Pulmonary vein stenosis is characterized by neointimal lesion formation that can be limited to the extraparenchymal veins or extend into the intraparenchymal veins. Human histological specimens demonstrate neointimal lesions that have myofibroblast-like cells and loose extracellular matrix deposition. Spindle-shaped cells or myofibroblast-like cells stain for alpha smooth muscle actin and vimentin, and extensive fibro-myxoid deposition in the mature lesions that stain strongly with Alcian Blue [12]. There is typically a paucity of inflammatory cells, while proliferating cells, using Ki-67 immunostaining, are modestly seen in less mature lesions [12,13]. In addition, activation of receptor tyrosine kinase receptors such as platelet-derived growth factor and fibroblast growth factor have been noted in histological specimens from children with PVS [14]. Of interest, a single case series has described the presence of ‘metakaryotic cells’, a type of stem cell in PVS lesions, and proposed that they may be a source of progenitor cells that contribute to fibroblast deposition [15]; however, no additional studies have verified these findings to date. Animal models support neointimal lesion formation with myofibroblast-like deposition as an important mechanism of PVS progression. Using a cut and sew model mimicking post repair PVS, the authors demonstrated a proliferative model whereby lesions stain positive for Ki67, a proliferative marker, and biochemical evidence of activated mTOR signaling [16]. In a neonatal bilateral banding model of PVS, myofibroblast lesions develop and progress into the intraparenchymal veins [17]. In this animal model, there is evidence supporting proliferation and endothelial-to-mesenchymal transition (EndMT) with elevated transforming growth factor-beta signaling that could be ameliorated with the use of losartan [18]. 

**Figure 2:**
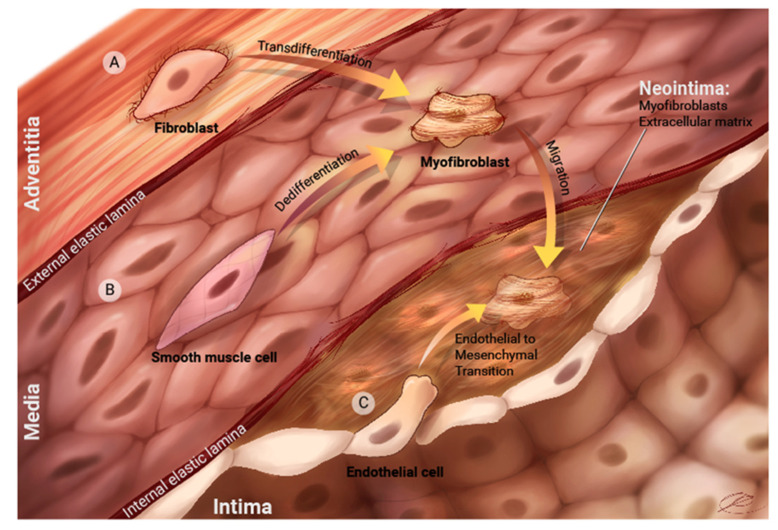
Potential sources of myofibroblast-like cells in pulmonary vein stenosis. Potential sources of cells could be through proliferation of existing pericytes and fibroblasts in the various vascular compartments (**A**), dedifferential of smooth muscle cells (**B**), endothelial-to-mesenchymal transition (**C**), and circulating progenitors cells. (Reprint permission, RD Vanderlaan, PVS Network, www.PVSNetwork.org, accessed on 1 May 2021).

The origin of the myofibroblast-like cells is currently an area of investigation and is an area where many potential therapeutic adjuncts are emerging. Myofibroblast cells that characterize neointimal lesions can come from many different sources and may have temporal contributions over different stages of lesion development (Figure 2). Potential sources of cells could be through proliferation of existing pericytes and fibroblasts in the various vascular compartments, endothelial-to-mesenchymal transition, dedifferentiation of smooth muscle cells and circulating progenitor cells. 

One hypothesis was that neointimal lesions represented a neoplastic-like process; however, an initial pilot study with methotrexate and vincristine did not improve survival [19]. In addition, analyses of pathological samples from children with PVS do not reveal a relationship with markers of pediatric fibroproliferative disorders, such as MHY9-USP6 translocation [13]. Losartan as a target of EndMT has been applied clinically, and data on a small cohort of patients who were treated with losartan as a medication to suppress EndMT are anticipated in 2022. Extending the antiproliferative effects of drug-eluting stents, systemic sirolimus has been used as an antiproliferative to mitigate in-stent stenosis in a single center retrospective series [20]. Use of imatinib, a tyrosine kinase inhibitor, and bevacizumab, an inhibitor of vascular endothelial growth factor, to target the upregulated tyrosine receptors seen in histological specimens has been shown to improve survival compared to the methotrexate and vincristine cohort [21]. While retrospective data on this cohort did not show a difference in overall survival compared to an aggressive multimodal surgical and catheter-based treatment cohort [7], use of propensity matching from a contemporary cohort could help refine our understanding of the incremental effects of these medical adjuvants. 

While primary and post repair PVS may have different inciting events, the progression and histological appearance of the disease appear more undifferentiated. Understanding the contribution of these cellular responses as the disease progresses over time will help target various therapeutic agents across the disease journey that can aid in slowing the progression of the disease, regardless of the inciting event. 

### 3.2. Modifying and Contributing Factors in PVS 

**Figure 3:**
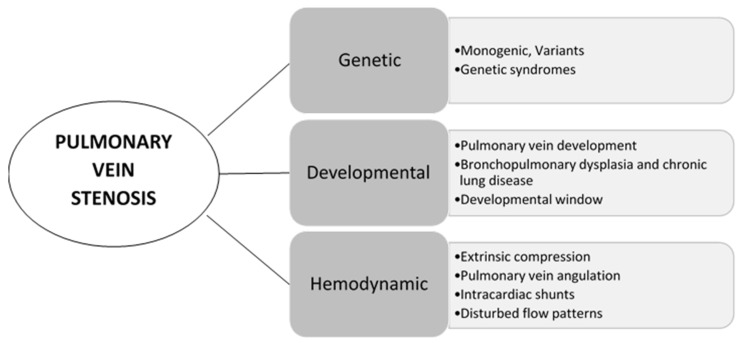
Modifying and contributing factors in pulmonary vein stenosis.

The etiology of PVS has remained elusive and likely has a multifactorial contribution. Likewise, the progression and trajectory of pulmonary vein stenosis may be affected by multiple modifying or contributing factors (Figure 3)**.**

#### 3.2.1. Genetic Contribution

In congenital or primary PVS, identification of a monogenic etiology has not been reported, unlike pulmonary arterial hypertension (PAH) or pulmonary veno-occlusive disease (PVOD), where familial inheritance has led to genetic candidates [22,23]. One case report documented a familial occurrence in consanguineous parents, without a candidate gene [24]. In families with anomalous drainage of the pulmonary veins, genetics studies identified PDGFRA and ANKRD1 [25,26], while whole genome sequencing studies found rare variants that require further investigations in larger cohorts [27]. SEMA3D was identified in a case of partial anomalous pulmonary veins which was recapitulated in mouse models [28]. However, none of these cohorts involved patients with stenosis and to date, no monogenic cause for PVS has been identified. Genetic syndromes occur in about 30% of patients, with Trisomy 21 being overrepresented [6,10]. Accelerated pulmonary hypertension is associated with T21, and a recent publication demonstrated that Trisomy 21 patients who had longer exposure to left to right shunts had increased risk of developing PVS [29]. Collaborative studies using whole genome sequencing of large cohorts are a priority and will use the existing PVS Network infrastructure. These studies have the potential to provide insight into modifying and contributory signaling pathways and gene networks important in progressive pulmonary vein stenosis.

#### 3.2.2. Developmental Contribution

Pulmonary vein development is a complex interplay between the lung bud and cardiac development [30] and in humans, while controversy exists, data support a single common pulmonary vein arising from the splanchnic mesoderm and incorporating with the dorsal mesenchyme of the left atrium, which is derived from the second heart field, to give rise ultimately to four separate pulmonary veins [30,31]. Extraparenchymal pulmonary veins are notable for the presence of a myocardial sleeve, which is an outer layer of cardiomyocytes, which surrounds the proximal pulmonary vein. While the etiology of PVS is unknown, one could postulate that venoatrial discrete narrowing or pulmonary vein atresia could arise from alterations in developmental programs during incorporation into the left atrium or branching of the common pulmonary vein into four distinct pulmonary veins. Alternatively, abnormalities of the myocardial sleeve or the cardiomyocytes that make up the sleeve could also lead to venoatrial narrowing through contraction or distortion. Pulmonary vein stenosis seems to have a developmental window for which the disease seems to be most aggressive. Indeed, younger age at diagnosis and weight less than 3 kg at time of repair have been identified in some series [4,6,7]. Typically, PVS is diagnosed within the first 6 months of life and disease occurring after the age of 3 years can be more indolent in its course. It is unclear if this is a disease that burns out due to overwhelming fibrosis and loss of proliferative cells or if it is a consequence of the evolving postnatal lung microenvironment.

In ex-premature infants with bronchopulmonary dysplasia (BPD), the development of pulmonary vein stenosis is an important comorbidity and can occur in about 30% of infants with BPD. In some case series, it has been associated with increased mortality [2,32], although other studies have not shown a clear association [33]. Development of PVS in this population is thought to be idiopathic and the contributory role of intracardiac shunts, hypoxia, necrotizing enterocolitis and mechanical ventilation on the premature pulmonary venous endothelium is not yet well defined [32,34].

#### 3.2.3. Intimal Trauma and Hemodynamic Contribution

Exposure to shunts and high flow have been postulated to play a role in the pathogenesis of PVS, as many children have atrial and ventricular shunts or patent ductus arteriosus. Most recently, in T21 children, exposure to shunts was associated with increased risk of PVS [29]. Extra-anatomical factors can also contribute to the development of PVS. Kotani and colleagues reported increased obstruction of left-sided veins in single ventricles related to cardiomegaly and anteriolateral displacement of the pulmonary veins [35,36]. Angulation of the pulmonary veins at the level of the pericardial reflection is also postulated to potentially create localized flow disturbances, and it is speculated that this may be exacerbated in ventilator-dependent premature children with regional atelectasis [37].

Turbulent flow is associated with abnormal intimal fibrosis and surgical techniques have evolved to minimize intimal trauma from suturing and disturbed flow patterns due to distortion when repairing pulmonary veins. The ‘sutureless’ technique was introduced to mitigate direct suturing on the pulmonary veins, which is thought to exacerbate tendencies for intimal fibrosis [1]. It is also thought to provide a more open egress of blood flow compared to conventional repair techniques by promoting laminar flow. Indeed, data are emerging that support the use of the sutureless technique in primary repair of total anomalous pulmonary veins as it is associated with decreased occurrence of post repair PVS [38,39,40].

Anatomic-based repair is an approach which takes into consideration the pathway of the individual pulmonary veins as they travel from the lung to the back of the left atrium [37] and attempts to provide unobstructed blood flow. All efforts are made to release pericardial tethering, resect thickened tissue and to shorten and straighten the course of the pulmonary vein, while using patch material to promote unobstructed laminar flow to the left atrium. Additionally, attention to the proximity of the atrial septum to the pulmonary vein ostia is important. In repair of pulmonary vein stenosis, shifting the position of the atrial septum away from the pulmonary vein ostia will potentially minimize turbulent flow at the ostia if septal malposition is present. Understanding the impact of various surgical approaches that minimize residual gradients and maximizing laminar flow will be of interest in PVS Network registry studies, as these can potentially influence outcomes.

## 4. Conclusions

PVS is a heterogenous disease that is evolving in its management. Multi-institutional PVS Network registry studies to improve clinical resolution of the disease are ongoing and will help to power prospective studies moving forward. Use of translational approaches such as the PVS Network genetics and biomarker studies will expand our knowledge, and likewise, integration of basic science to address clinically relevant questions will further our understanding and aid in finding medical therapies to slow the progression of this disease. Through collaboration, incremental knowledge gains will translate into improved outcomes for children diagnosed with PVS.
